# Transcriptome-Wide Association Study of Metabolic Dysfunction-Associated Steatotic Liver Disease Identifies Relevant Gene Signatures

**DOI:** 10.5152/tjg.2024.24326

**Published:** 2024-12-23

**Authors:** Jianxiu Wang, Qian Gao

**Affiliations:** 1 Department of Emergency Medicine, Shandong University, Qilu Hospital (Qingdao), Cheeloo College of Medicine, Qingdao, China

**Keywords:** MASLD, GWAS, TWAS, FUMA, fine-mapping, SMR

## Abstract

**Background/Aims:**

Metabolic dysfunction-associated steatotic liver disease (MASLD) is considered the most widespread chronic liver condition globally. Genome-wide association studies (GWAS) have pinpointed several genetic loci correlated to MASLD, yet the biological significance of these loci remains poorly understood.

**Materials and Methods:**

Initially, we applied Functional Mapping and Annotation (FUMA) to conduct a functional annotation of the MASLD GWAS summary statistics, which included data from 3242 cases and 707 631 controls. Additionally, a MASLD transcriptome association study (TWAS) was conducted utilizing FUSION software in combination with the genotype-tissue expression project (GTEx-v8) expression weight set to identify susceptibility genes. Furthermore, to elucidate the observed correlations, we carried out conditional and joint analyses, probabilistic causal fine-mapping of TWAS signals, summary data-based Mendelian randomization (SMR), and phenome-wide association analyses.

**Results:**

Following functional annotation analysis, we identified 4 genetic risk loci, annotated 6 lead single nucleotide polymorphisms (SNPs), 27 independent significant SNPs, and 511 candidate SNPs. TWAS also found four genes related to MASLD, including MAU2 sister chromatid cohesion factor (MAU2), EPH receptor A2 (EPHA2), GATA zinc finger domain containing 2A (GATAD2A), and transmembrane 6 superfamily member 2 (TM6SF2). Moreover, fine mapping of TWAS signatures identified 13 causal genes associated with MASLD that were located at 3 genetic risk loci, but SMR results could not rule out the possibility that the relationship between significant genes and MASLD was caused by a linkage disequilibrium structure.

**Conclusions:**

Our study found new significantly associated genes for MASLD and highlighted the ability of TWAS to identify and prioritize potentially pathogenic genes.

**Main Points**

- Functional annotation, gene-based analysis, and gene-set analysis of summary data from genome-wide association studies (GWAS) on metabolic dysfunction-associated steatotic liver disease (MASLD) revealed four genetic risk loci and highlighted eleven genes with Bonferroni-corrected significance.
- Transcriptome association study found four genes related to MASLD, including MAU2, EPHA2, GATAD2A, and TM6SF2.
- Summary data-based Mendelian randomization could not rule out the possibility that the relationship between significant genes and MASLD was caused by a linkage disequilibrium structure.

## Introduction

Metabolic dysfunction-related fatty liver disease (MASLD), formerly known as non-alcoholic fatty liver disease (NAFLD), is defined as the presence of at least one cardiometabolic risk factor and without harmful alcohol intake and patients’ health-related quality of life can be seriously affected.^1^ The MASLD encompasses a spectrum of liver diseases that are simple hepatic steatosis, fibrosis, metabolic dysfunction-related steatohepatitis (MASH, previously NASH), cirrhosis, and MASH-related hepatocellular carcinoma.^2^ It is closely related to the characteristics of metabolic syndrome, including insulin resistance, obesity, type 2 diabetes mellitus (T2DM), and dyslipidemia.^3^ According to global statistics, MASLD has a significant prevalence of about 30%,^4^ making it the most important chronic liver disease worldwide, and the global burden of liver disease is increasing.^5^ Due to the etiology’s complexities, the precise pathophysiology of MASLD is unknown. Genetic susceptibility, dietary imbalances, and environmental factors may all contribute to MASLD.^6^ Human genome-wide association studies (GWAS) have significantly advanced our comprehension of the genetic underpinnings of MASLD progression, pinpointing numerous genetic loci strongly linked to the disease.

GWAS has recently demonstrated evidence of a connection between genetic variants (susceptibility loci *PNPLA3* and *TM6SF2*) and MASLD, which are less than a minority of the disease heritability.^7^ While GWAS have made significant strides in identifying factors contributing to the genetic architecture of MASLD, connecting these genetic loci to specific biological traits remains challenging. These studies frequently associate loci with neighboring genes, potentially introducing bias, particularly for longer genes, and may not fully elucidate the functional relevance of these genetic loci. In contrast, transcriptome-wide association studies (TWAS) utilize disease-related specific cell types and tissues, as well as databases with detailed records of tissue-specific expression, resulting in better interpretable biological effects.^8^ Integrating genetic and transcriptional variation through a targeted approach using smaller reference populations can effectively identify expressed genes associated with complex traits in MASLD GWAS datasets.

To identify genes associated with MASLD, we performed TWAS using the latest MASLD GWAS summary statistics. Moreover, fine-mapping, summary data-based Mendelian Randomization (SMR), and phenome-wide association studies were conducted to characterize genes associated with the risk of MASLD.

## Materials and Methods

The MASLD GWAS summary data used in this study are from UK Biobank and Finngen databases, which are public databases. The patients involved in the database have obtained ethical approval. Researchers are permitted to download relevant data for free for scholarly purposes and publish relevant articles. Our investigation relies on open-source data, follows the guidance of ethics committees, and is free from ethical issues and other conflicts of interest.

### Functional Mapping and Annotation (FUMA) Based on GWAS

The FUMA (v1.4.1) pipeline, which supported the human genome hg19, annotated the results of GWAS summary statistics based on SNPs’ functional and positional information. Briefly, the genomic loci showing significant association with the trait were delineated utilizing GWAS summary statistics and the linkage disequilibrium structure derived from the 1000 Genomes Project, focusing on the European population. Lead SNPs and candidate SNPs were identified using the criteria listed below: (i) independently significant SNPs were those with *P*-values < 5e−08 and independent from each other with r^2^ < 0.6; (ii) candidate SNPs were defined as having r^2^ ≥ 0.6 with one of the independently significant SNPs, a minor allele frequency > 0.01, and were selected for further annotation; (iii) independent lead SNPs were identified as independently significant SNPs that were also independent from each other with r^2^ < 0.1. Genomic risk loci were identified by aggregating significant SNPs within a 250 kb window and all SNPs with r^2^ ≥ 0.6 in linkage disequilibrium with one of the independently significant SNPs.^9^

### Multi-marker Analysis of GenoMic Annotation (MAGMA) for Gene-Based and Gene-Set Analysis

P-values for gene-based gene set analysis were calculated utilizing MAGMA (v1.08), integrated within FUMA. In the gene-based analysis, P-values were computed based on protein-coding genes if the GWAS results indicated that SNPs were located within these genes. The SNP-wide mean model and 1000 Genome Phase 3 reference panels were implemented in gene analysis. Furthermore, we detected prioritized genes from a total of 18 883 genes from Ensemble v92 with a Bonferroni-adjusted *P*-value of 0.05/18 883 = 2.65e−06 for gene-based analysis. Gene-set *P*-values were computed utilizing 10 678 gene sets sourced from MsigDB v5.2, comprising curated gene sets (4761) and Gene Ontology (GO) terms (5917), with a Bonferroni-corrected significance threshold of 8.4e−06.

### Transcriptome-Wide Association Studies

To uncover candidate genes linked to MASLD risk, we compiled MASLD GWAS summary data from the UK Biobank study (1664 cases and 400 055 controls of European ancestry) and the FinnGen consortium (1578 cases and 307 576 controls of European ancestry).

TWAS analysis using FUSION was conducted to identify genes potentially influenced by gene-regulated expression associated with MASLD risk. Considering tissue-specific gene expression and LD structure between SNPs, GTEx (v8) reference panels (liver and whole blood) were used to derive expression weights using prediction models integrated into FUSION (http://gusevlab.org/projects/fusion/#gtex-v8-multi-tissue-expression). Predictive models such as BLUP, LASSO, BSLMM, Elastic Net. and top SNPs were employed in FUSION to generate expression weights for the reference sets.^8^ A strict Bonferroni corrected threshold was applied: *P *= .05/12 926 (3.87e−06) (total number of genes in the reference panels).

### Bayesian Colocalization

To assess colocalization between GWAS SNPs and eQTLs, we integrated summary statistics from MASLD GWAS with liver and whole blood eQTL results using the COLOC package in R. COLOC was executed under five hypotheses: H0 (no eQTL or GWAS association), H1 (association with eQTL but not GWAS), H2 (association with GWAS but not eQTL), H3 (independent eQTL and GWAS signals), and H4 (shared eQTL and GWAS associations). The outcome of this analysis yields five posterior probabilities (PP0, PP1, PP2, PP3, and PP4).^10^ The primary objective was to determine if the GWAS and eQTL signals showed evidence of shared causal variants. Furthermore, a high posterior probability (PP4 > 80%) indicated colocalization between the GWAS and eQTL signals.^11^

### Joint-Conditional Tests

Conditional tests were conducted to assess if numerous significant features within a locus indicate independent connections or to quantify the residual GWAS signal after accounting for TWAS expression associations.^8^ We employed FUSION to calculate genome-wide Bonferroni-corrected TWAS signals, and residual associations of SNPs with MASLD were assessed to collectively quantify the impact of all significant features within each locus.

The identified regions encompass only the transcriptional domains of genes, with each connection of MASLD GWAS SNPs conditioned on a comprehensive gene model, evaluated one SNP at a time.^12^

The conditional analysis identified which traits exhibited independent associations and which lost significance after accounting for the projected expression of other traits within the domain.^13^

### Probabilistic Fine M apping of TWAS

TWAS predicted expressions led to significant gene-trait associations, including non-causal genes influenced by linkage disequilibrium and SNP pleiotropy. Fine Mapping of Causal Gene Sets (FOCUS), utilizing GWAS summary statistics, expression quantitative trait locus weights, and SNP linkage disequilibrium structure as inputs, estimated the likelihood of TWAS association signals.^14^ FOCUS can obtain posterior inclusion probabilities (PIP) of causal genes from expression predictions of other relevant tissues. FOCUS yielded 90% confidence credible sets for significantly associated causal genes, which were applied to functional prediction and prioritization of risk genes. PIP values for individual traits, with a value > 0.5, indicate that a trait is more prone to be causative than any other trait in the related region.^13^ FOCUS for fine-mapping features is implemented in all SNP reference panels, independent of tissue priority.

### SMR-HEIDI

We utilized a Summary data-based Mendelian Randomization (SMR) analysis to investigate the pleiotropic correlation between MASLD SNPs and gene expression. In brief, this technique combines GWAS and eQTL studies to infer causal variation in gene expression-trait relationships using MR principles. A total of three eQTL studies were applied to our SMR analysis. The first study includes local effects summary statistics from the GTEx v8 project across the liver and entire blood tissues.^15^ The second was the largest eQTL meta-analysis hitherto reported by *Westra et al*, involving peripheral blood samples from 5311 European populations.^16^ The third dataset utilized was sourced from the Consortium for the Architecture of Gene Expression (CAGE), comprising genotype and whole blood expression data from 2765 individuals of European descent.^17^ In the eQTL summary statistics, variants with a minor allele frequency < 0.01 were excluded to mitigate potential false positives arising from linkage disequilibrium. For SMR analysis, we employed significant probes with a strict Bonferroni-corrected SMR *P*-value threshold (0.05/number of probes). The heterogeneity of correlation instrument (HEIDI) test was conducted to assess whether there was causality rather than linkage disequilibrium affecting gene expression in the observed correlations.^18^ The HEIDI test was the null hypothesis for MR analysis, and those probes with little heterogeneity difference (*P*_HEIDI_ ≥ 0.05) were preserved.

### Phenome-Wide Association Studies

A phenome-wide association study was performed for each SNP to locate the traits related to the top eQTL for each TWAS gene. The top ten traits (excluding MASLD) were reported. The GWAS Atlas public data was used in the phenome-wide association study (https://atlas.ctglab.nl/).

## Results

### Functional annotation analysis of FUMA

**Figure 1.**
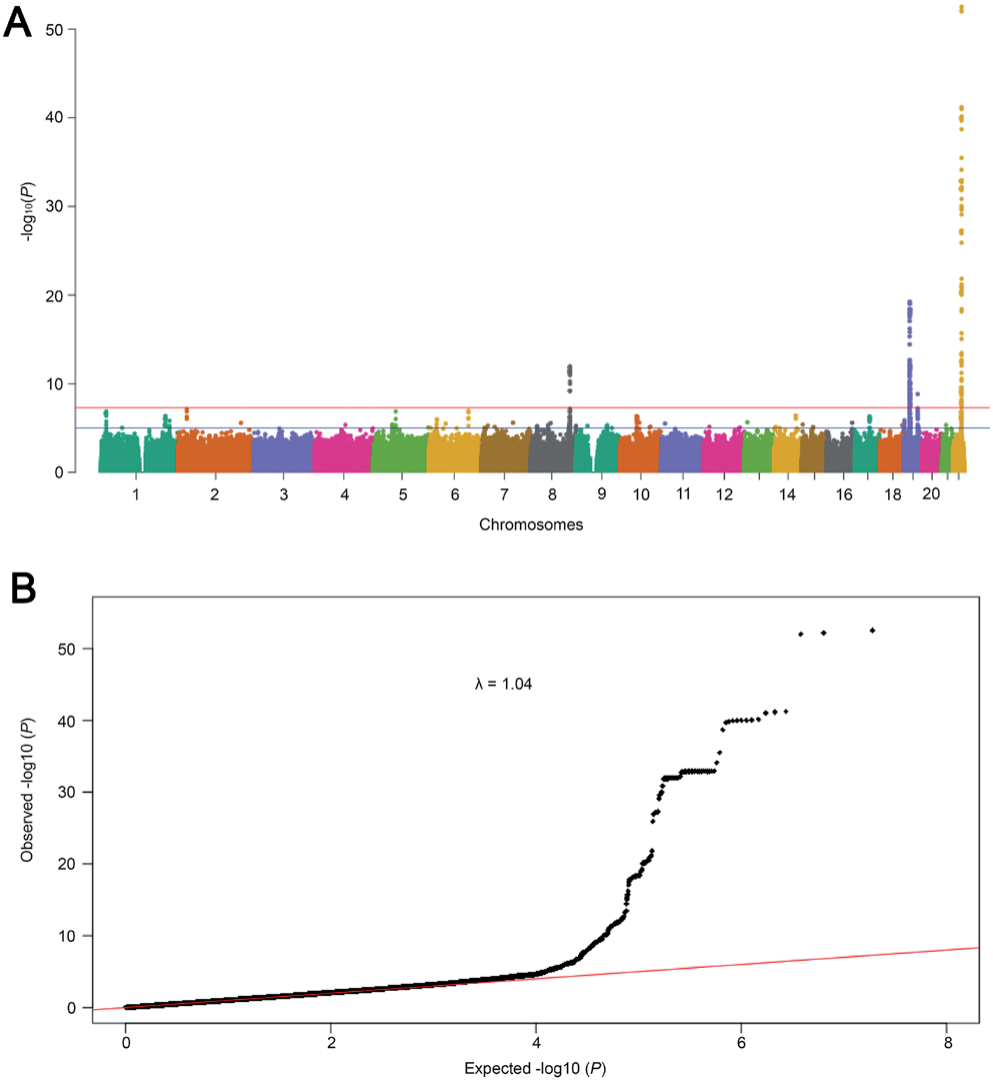
Manhattan plot and quantile-quantile plot of the genome-wide *P* values in the GWAS analysis. (a) The Manhattan plot showed the genome-wide association statistics from the GWAS analysis. The *x*-axis represents the genomic position, and the *y*-axis shows the −log_10_ (*P*). The red dashed line indicates the genome-wide significance threshold of *P* = 5e−08. (b) The quantile-quantile plot. The red line represents the null hypothesis of no true association. The black dot with gradient λ (inflation coefficient) is fitted to the lower 90% of the distribution of the observed test statistics. The value of the inflation factor is 1.04.

**Figure 2.**
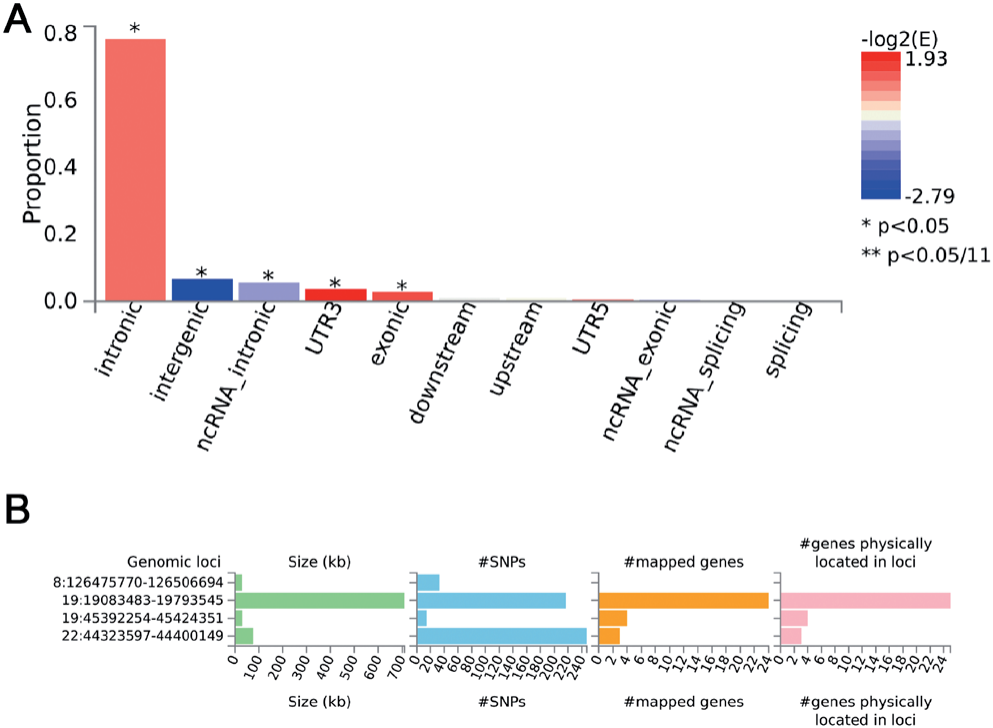
Functional mapping and annotation based on genome-wide association study. (a) Functional consequences of SNPs on genes. Enrichment of functional consequences of SNPs was tested against the reference panel population of the 1000 Genomes Phase 3 European. All SNPs that are in LD with one of the independent significant SNPs are annotated by FUMA (https://fuma.ctglab.nl/). Enrichment value was computed as (proportion of SNPs with an annotation)/(proportion of SNPs with an annotation relative to all available SNPs in the reference panel). Fisher’s exact test (2 sides) was performed for each annotation as above, and then the enrichment levels were log2 transformed. (b) Summary per genomic risk locus. After functional annotation analyses, we annotated 511 candidate SNPs that passed the gene-wide significance threshold (*P* < 5e−08), 6 independent lead SNPs were identified located at 4 genomic risk loci. kb, kilobase; ncRNA, non-coding RNA; SNP, single-nucleotide polymorphism.

We used the web FUMA platform with MASLD GWAS summary statistics as input files for functional annotation. FUMA (v1.4.1) identified four significant genomic risk loci associated with MASLD (Supplementary Table 1). Six lead SNPs, 27 independent significant SNPs, and 511 candidate SNPs were identified among these loci (Supplementary Tables 2-4). The MASLD GWAS summary statistics were used to generate a Manhattan plot (Figure 1a). The quantile-quantile plot demonstrated strong concordance between observed and predicted P-values, suggesting that the GWAS summary statistics were well-calibrated (λ = 1.04) (Figure 1b). FUMA detected independently significant SNPs in intronic regions with significant enrichment (78.3%), while only 1.87% were identified in intergenic, non-coding RNA intronic, UTR3, and exonic regions, respectively (Supplementary Table 5, Figure 2).

### Gene-Based and Gene-Set Analyses in MAGMA

**Figure 3.**
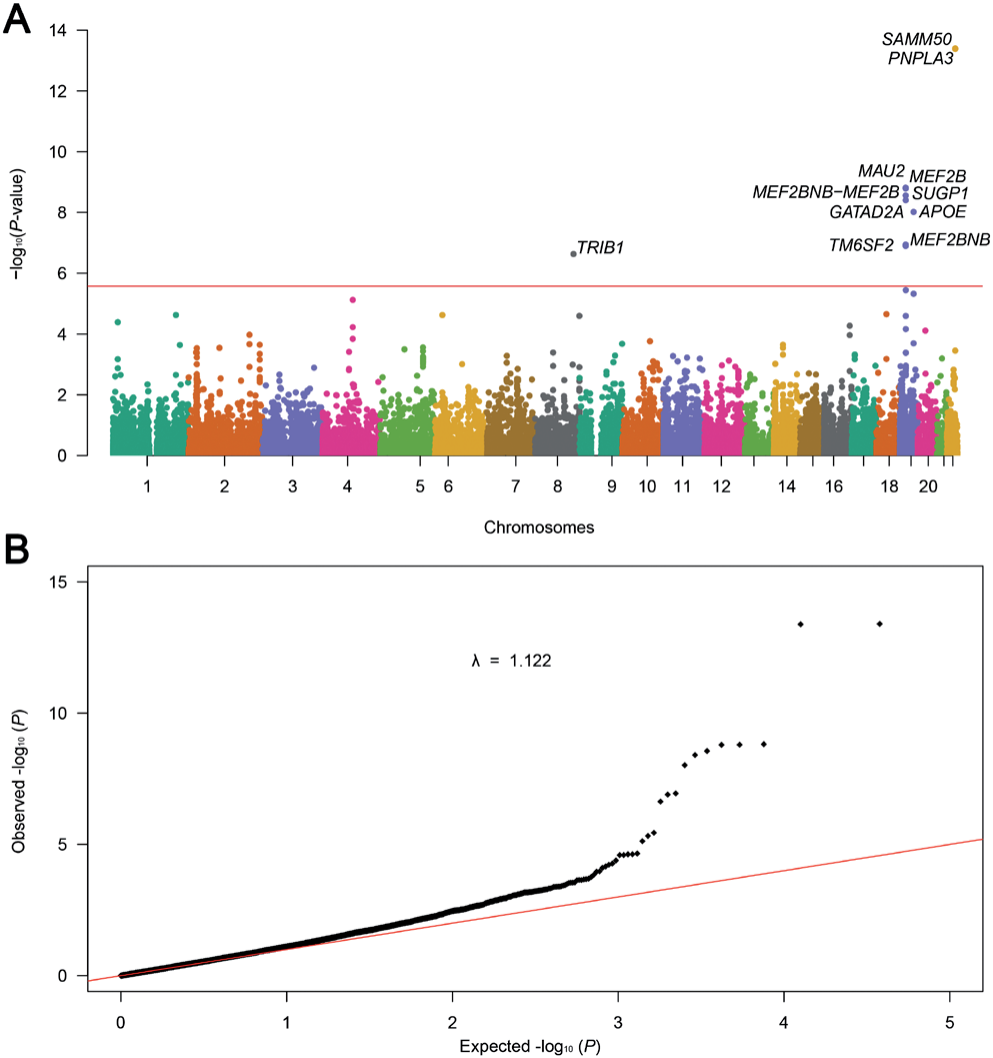
Gene-based genome-wide analysis for MASLD by MAGMA to each locus in the GWAS analysis. (a) Significant genes in the gene-based association test in MAGMA after Bonferroni correction (*P *< .05/18 887 = 2.65e−06). The *x*-axis represents the chromosome number, and the *y*-axis shows the negative log10-transformed gene-based *P*-value. The top 11 most significant genes are annotated with the corresponding gene symbols. (b) The quantile-quantile plot. The red line represents the null hypothesis of no true association. The black dot with gradient λ (inflation coefficient) is fitted to the lower 90% of the distribution of the observed test statistics. The value of the inflation factor is 1.122.

The gene-based analysis for MASLD summary data detected 11 prioritized genes (*SAMM50*, *PNPLA3*, *MEF2BNB-MEF2B*, *MEF2B, MAU2, SUGP1, GATAD2A, APOE, TM6SF2, MEF2BNB,* and *TRIB1*) with Bonferroni adjustment for significance at *P* < 2.65e−06 (Figure 3, Supplementary Table 6). The gene-set analysis discovered the following top genes ontology pathways: “fibroblast growth factor activated receptor activity” (category: molecular function, *P*-value = 3.39e−06); “Ouellet cultured ovarian cancer invasive vs lmp dn” (category: curated gene sets *P*-value = 2.18e−05); “eosinophil differentiation” (category: biological processes *P*-value = 3.11e−05); “plasma lipoprotein particle clearance” (category: biological processes, *P*-value = 3.81e−05); and “flavonoid glucuronidation” (category: biological processes *P*-value = 4.01e−05) (Supplementary Table 7).

### Identification of Significant Genes of MASLD by TWAS

**Figure 4.**
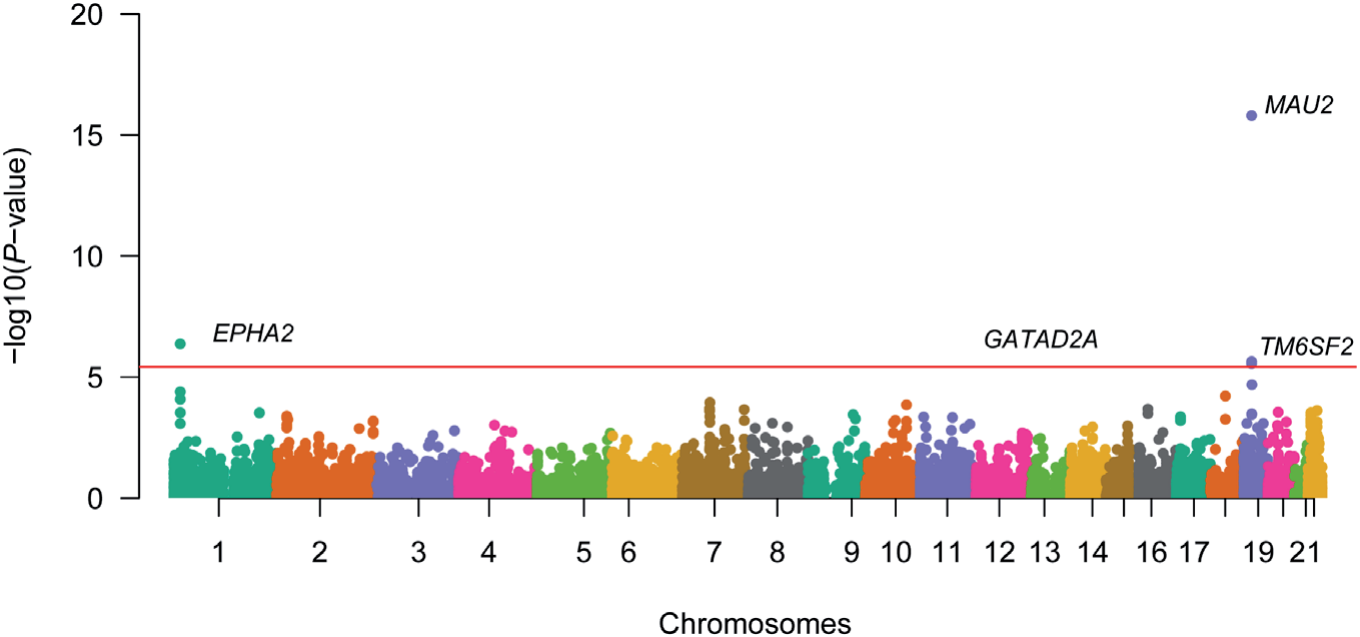
Manhattan plot of the transcriptome-wide association study for MASLD (n = 3242 cases and n = 707 631 controls). Bonferroni-corrected significant genes are labeled. A significance threshold of *P* = 3.87e−06 was used.

The TWAS processes were analyzed employing the MASLD GWAS summary data with liver and whole blood eQTL data sets. Four genes were discovered to be closely correlated to MASLD (Figure 4 and Table 1).

The gene EPHA2, situated in the p36.13 region of chromosome 1, exhibited transcriptome-wide significance (TWAS *P-*value = 4.25e−07) and represented the sole significant correlation signal at this locus (Table 1). An intergenic variant, rs6677710, showed the strongest association with MASLD (odds ratio [OR] = 0.87, PGWAS = 1.87e−07) within this locus. Moreover, rs7538216, which is in strong linkage disequilibrium with rs6677710, was identified as the primary eQTL affecting the EPHA2 gene expression level (*P*_eQTL_ = 2.7e−09). (*r*^2^ =1). (https://pubs.broadinstitute.org/mammals/haploreg/haploreg.php). Subsequently, formal Bayesian colocalization analysis revealed a shared signal with a posterior probability (PP4) of 0.99 (Table 1), confirming that the significant MASLD GWAS signal and the liver eQTL signal were driven by the same causal variants.

Three transcriptome-wide important genes (*MAU2*, *GATAD2A*, and *TM6SF2*) were identified within the p13.11 region of chromosome 19 (TWAS *P* value = 1.57e−16, 2.29e−06, and 2.84e−06, respectively) (Table 1). The intronic variant rs12610185 exhibits the strongest correlation with MASLD at its respective locus (OR =1.50, *P*_GWAS _= 3.83e−19). Meanwhile, we identified that the top eQTLs in the locus—rs859287, rs808203, and rs4808200—were correlated with the expression levels of the MAU2, GATAD2A, and TM6SF2 genes in liver and whole blood tissues (*P*_eQTL_ = 6.2e−14, 2.2e−29, and 1.19e−04, respectively). In addition, colocalization analysis revealed PP4 values indicating causality (MAU2, PP4 = 0.98; GATAD2A, PP4 = 0; and TM6SF2, PP4 = 0.39), suggesting that the significant MASLD GWAS signal (MAU2) and whole blood eQTL signals share common causal variation at their respective loci.

**Figure 5.**
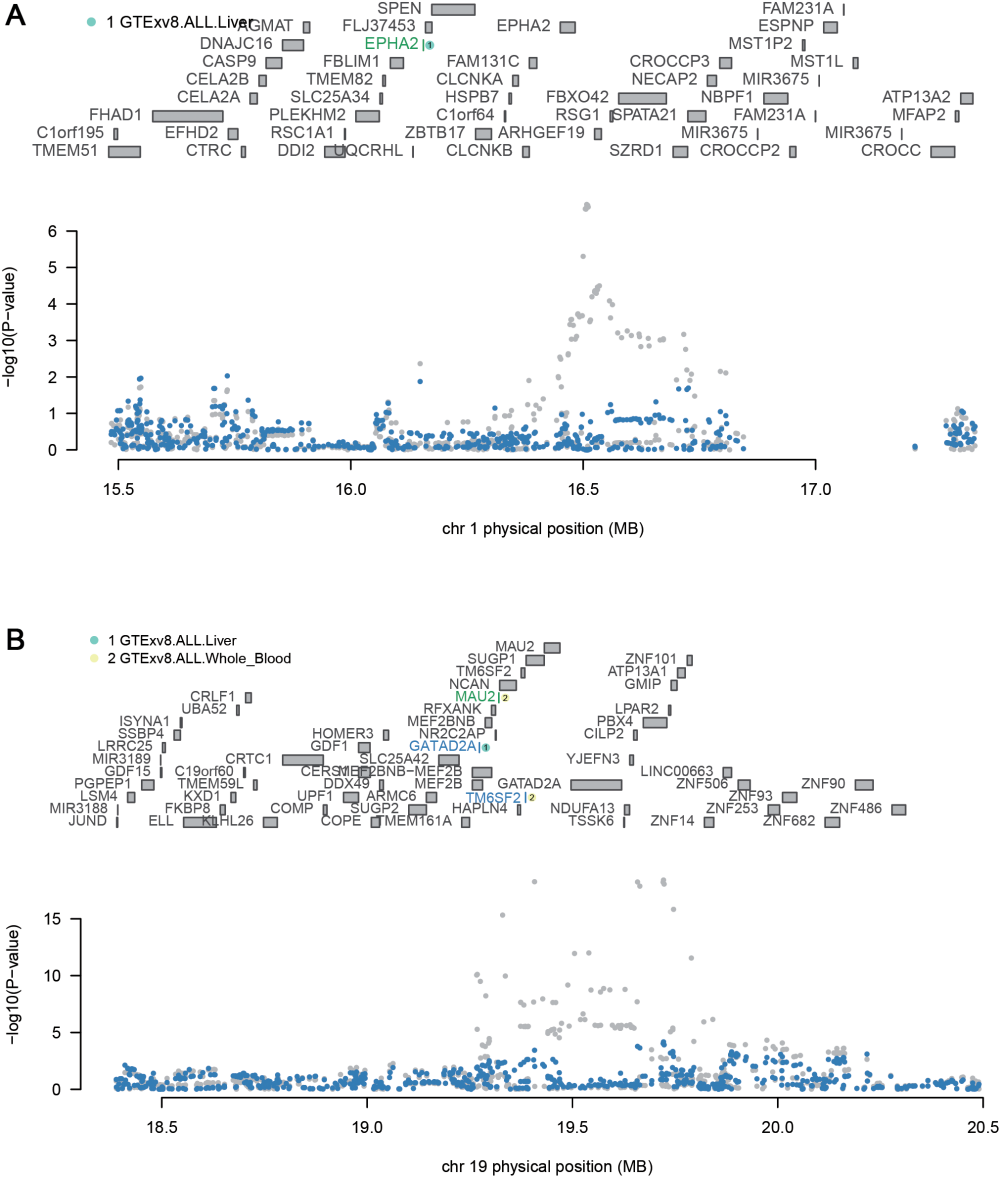
Regional association of TWAS hits. (a). Chromosome 19 regional association plot. (b) Chromosome 1 regional association plot. The top panel in each plot highlights all genes in the region. The marginally associated TWAS genes are shown in green and the jointly significant genes are shown in blue. The bottom panel shows a regional Manhattan plot of the GWAS data before (gray) and after (blue) conditioning on the predicted expression of the blue genes.

Given the overlap of multiple TWAS genes with significant MASLD loci, joint and conditional tests were conducted to ascertain their conditional independence. Results indicated that conditioning on EPHA2 fully accounted for the signal observed at the loci on chromosome 1 (rs6677710 lead SNP GWAS *P *= 1.87e−07, conditioned on *EPHA2* lead SNP *P*_GWAS_ = 1) (Figure 5a). Conditioning on *MAU2* explained 0.801 variances of the loci on chromosome 19 (rs12610185 Lead SNP *P*_GWAS_ = 3.83e−19, lead SNP *P*_GWAS_ = 6.70e−05) (Figure 5b).

### Fine Mapping of TWAS Signals

To select responsible genes for expression prediction, FOCUS was used to fine-mapping posterior probabilities for genes in TWAS signals and associated tissues. We discovered 13 unique genes (absolute value of TWAS |Z-score| > 6) that may be directly correlated to MASLD (Table 2). For the gene loci, 22:43714200-22:44995308, *PNPLA3* and *TTLL12* were contained in the 90% credible gene set. In the skin sun-exposed lower leg and lung, PNPLA3 had the highest posterior probability as the causal gene at 0.87, while in the small intestine terminal ileum, TTLL12 showed a posterior probability of 0.45. For the gene locus 8:126410917-8:128659111, LINC00964 and TATDN1 were among the credible genes. The posterior probability of causation for LINC00964 in the heart atrial appendage was 0.54, whereas TATDN1 in the muscle-skeletal system was 0.45. The credible gene set for loci 19:18409862-19:19877471 comprised GATAD2A, ZNF93, ZNF90, LINC00663, ATP13A1, NCAN, TM6SF2, MAU2, and ZNF486. Except for *GATAD2A* (for 0.65 in the esophagus mucosa) and *ZNF93* (for 0.32 in the adrenal gland), the posterior probabilities of other risk genes were lower (Table 2).

### SMR-HEIDI

**Figure 6.**
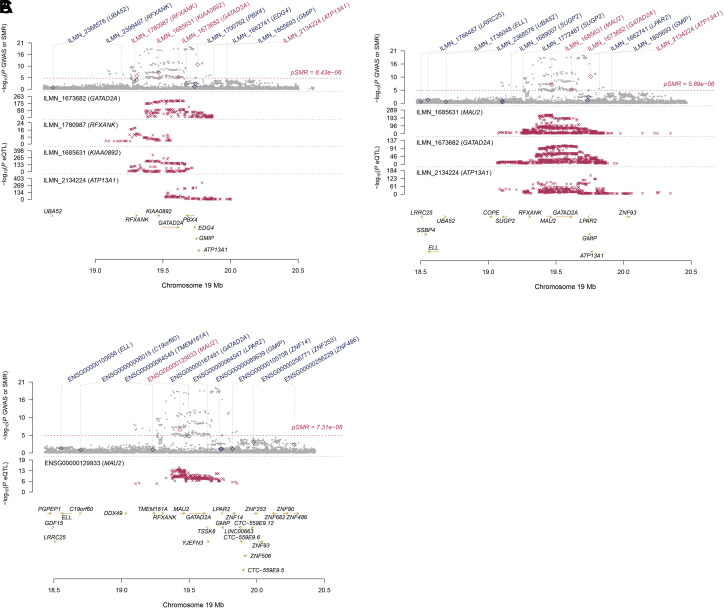
Important genes associated with MASLD identified in SMR analysis. The significant genes expression were directly associated with MASLD according to SMR analysis based on expression quantitative trait loci (eQTL) conducted by *Westra et al *(a), data from the CAGE study (b), and GTEx whole blood project (c), respectively.

**Figure 7.**
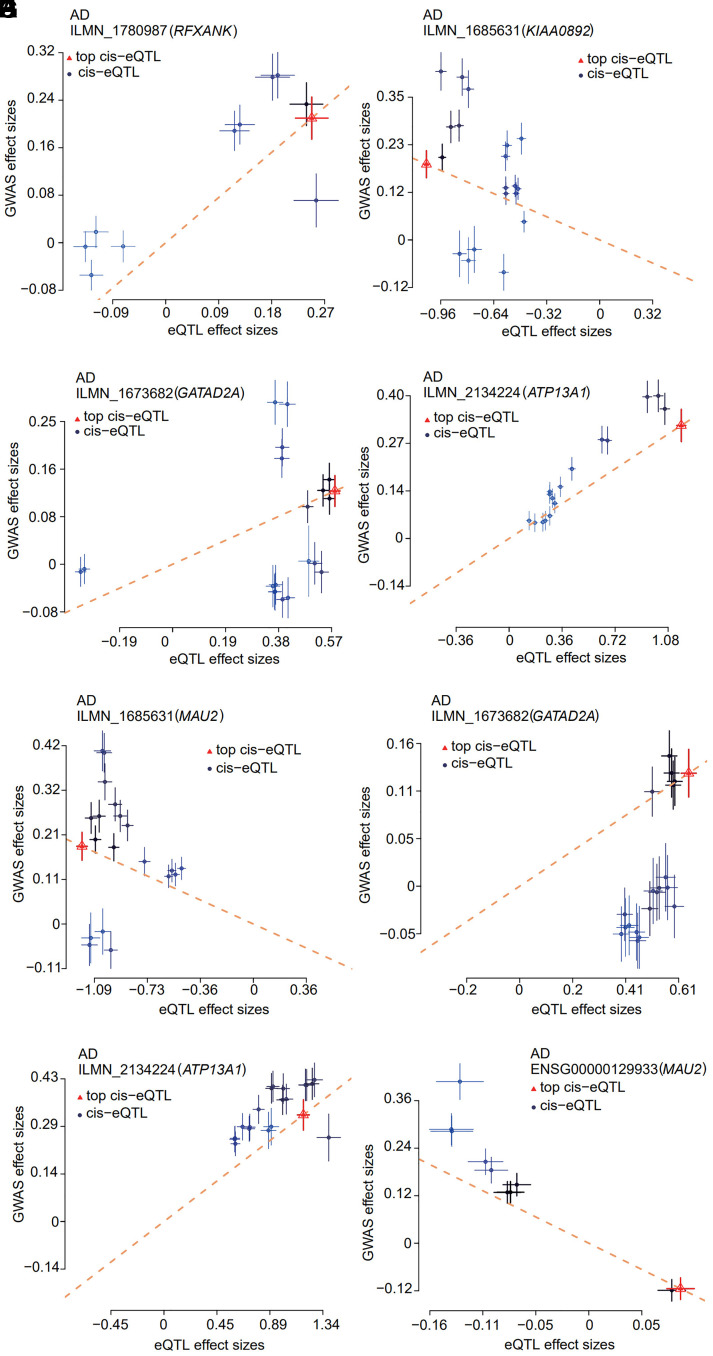
Identification of important genes for MASLD by SMR analysis. Effect sizes of SNPs (used for the HEIDI test) from GWAS plotted against those for SNPs from the Westra (a-d), CAGE study (e-g) and GTEx whole blood project (h). The orange dashed lines represent the estimate of *b_xy_* at the top *cis*-eQTL (rather than the regression line). Error bars are the standard errors of SNP effects.

Following the association of MASLD SNPs and three eQTL studies (GTEx, Westra, and CAGE), SMR analysis identified five significant genes on chromosome 19, including *RFXANK*, *KIAA0892*, *GATAD2A*, *ATP13A1*, and *MAU2 *(Table 3). We observed that the expression of the aforementioned significant genes showed direct associations with MASLD, as indicated by SMR analysis employing eQTL data from the CAGE study (*MAU2*: *P*_SMR_ = 4.19e−08; *GATAD2A*: *P*_SMR_ = 3.70e−06; *ATP13A1*: *P*_SMR_ = 1.89e−11, respectively), GTEx project (*MAU2*: *P*_SMR_ = 1.28e−07) and Westra study (*RFXANK*: *P*_SMR_ = 8.29e−07, *KIAA0892*: *P*_SMR_ = 2.88e−08, *GATAD2A*: *P*_SMR_ = 3.07e−06, *ATP13A1*: *P*_SMR_ = 16.62e−12, respectively) (Table 3, Figures 6 and 7). The above SMR analysis results suggest that SNPs for SMR-significant genes may affect MASLD by influencing the expression level of the five significant genes.

However, HEIDI tests were performed to distinguish eQTL SNP association patterns.

All five genes failed the HEIDI test (*P*_HEIDI_ < .05), indicating that the obtained remarkable association may be due to a high degree of LD between two different genetic variants (Table 3). Based on these data, we cannot dismiss the possibility that the correlation between MASLD SNPs and eQTL SNPs reflected by significant SMR results is due to an LD structure.^19^ Therefore, a larger sample size of MASLD GWAS and eQTL data is required to further elucidate these results.

### Phenome-Wide Association Studies

To explore phenotypes potentially correlated or co-morbid with MASLD, a phenome-wide association study was conducted on four significant genes identified through TWAS (Table 4). Since the phenome-wide association study screened for many traits correlated with MASLD, we emphasized the top ten most relevant items to be listed in Table 4. Several risk-related traits, such as triglycerides, total cholesterol, and low-density lipoprotein cholesterol, were revealed to be significantly correlated with key genes. These traits were previously recognized as risk factors for MASLD, reinforcing the correlation of significant genes.

## Discussion

MASLD is a persistent liver condition impacting millions of individuals worldwide, and its exact cause is unclear. In general, genetic predisposition to MASLD is pivotal in its etiology.^20^ In this study, we employed publicly accessible MASLD GWAS summary data from the UK Biobank and FinnGen consortium. Combining these datasets, our analysis comprised 3242 MASLD cases and 707 631 controls, representing the most extensive genomic investigation of MASLD clinical diagnosis to date. We subsequently analyzed the combined MASLD GWAS summary data using the bioinformatics tool FUMA for functional annotation, gene-based analysis, and gene set analysis. Four genetic risk loci were identified, and eleven Bonferroni-corrected significant genes were prioritized (*SAMM50*, *PNPLA3*, *MEF2BNB-MEF2B*, *MEF2B, MAU2, SUGP1, GATAD2A, APOE, TM6SF2, MEF2BNB,* and *TRIB1*), eight of which (*SAMM50*, *PNPLA3*,* MAU2, SUGP1, GATAD2A, APOE, TM6SF2, *and *TRIB1*) have been reported to exert roles in MASLD.^21,22^ Furthermore, gene set analysis identified MASLD-related pathways such as fibroblast growth factor-activated receptor activity, eosinophil differentiation, and plasma lipoprotein particle clearance. Additionally, the production of plasma lipoprotein particles, such as very low-density lipoproteins, has been linked to MASLD.^23^

Despite recent GWAS successfully identifying risk loci for MASLD, elucidating the functional implications of these associations remains challenging due to the complexity of pinpointing tissue-specific genes involved. We carried out a MASLD TWAS utilizing summary statistics from the MASLD GWAS. Using a publicly available genotype-expression reference panel, this approach supports attribution and correlation analysis of independent, large-scale data that used a machine learning approach.^24^ We discovered four genes linked to the risk of MASLD in the liver and whole blood, on chromosomes 1p36.13 and 19p13.11, respectively. One gene (EPHA2) has not been previously reported in the MASLD GWAS, while three genes (MAU2, GATAD2A, and TM6SF2) have been shown to be implicated in MASLD.^21,25^ Interestingly, the conditional and combined analyses showed that the TWAS expression signal contributed to the importance of the previously identified MASLD gene. Four significant genes exhibited two independent linkages with MASLD, suggesting that possibly half of the noted signal was affected by the LD or neighboring genes associated with predicted expression. Our TWAS results indicated that EPHA2 expression entirely explained the implicated MASLD GWAS signal, suggesting that TWAS has the ability to prioritize genes of interest. These results support the significant role of transcription in mediating the relationship between genetic susceptibility and MASLD. However, neither TWAS nor conditional analysis can definitively establish causality. In this study, we combined GWAS and eQTL data with MR analysis to look for promising genes with a potential causal connection to MASLD. We identified 5 significant genes from GTEx, Westra, and CAGE datasets. A recent genome-wide meta-analysis of MASLD in 4 cohorts of European ancestry participants with electronic health records showed that variant genes such as *MAU2* were negatively associated with MASLD-associated signature enzyme (alkaline phosphatase).^21^ In a comprehensive study involving liver biopsy-confirmed MASLD cases in Japan, genetic variants including GATAD2A were identified as significantly associated with an elevated risk of MASLD (*P* = 2.3e−08, OR (95%CI) = 1.37 (1.23–1.53)).^26^ However, the mutations of* RFXANK*, *KIAA0892,* and *ATP13A1* have not been reported to be associated with MASLD.

Methods for prioritizing causal genes, such as utilizing FOCUS, probabilistically aid in fine-mapping plausible candidates.^14^ In the process of fine mapping TWAS findings, PNPLA3 was identified as the top candidate, showing a posterior probability of 0.87 in the skin exposed to sunlight on the lower leg and lung tissues. Research has documented a stronger association between MASLD and lung cancer in women.^27^ The PNPLA3 I148M variant is recognized as a major inherited factor for MASLD, while PNPLA3 itself acts as an independent risk factor for hepatocellular carcinoma in individuals with MASH.^28^ At the other locus, LINC00964 and TATDN1 exhibited posterior probabilities of 0.54 and 0.45 in skeletal muscle and the terminal ileum of small intestines. Prior research indicates that long non-coding RNA TATDN1 promotes the proliferation of hepatocellular carcinoma.^29^ However, no known relationship has been established between LINC00964 and MASLD. In the third locus, GATAD2A had a posterior probability of 0.65 in the esophageal mucosa. GATAD2A was significantly increased in the oesophageal mucosa after long-term alcohol consumption, demonstrating a significant positive correlation with the severity of MASLD.^25^ Furthermore, despite the low posterior probability, other genes at this locus, such as ZNF93, ZNF90, LINC00663, ATP13A1, NCAN, TM6SF2, MAU2, and ZNF486, are included in the confidence set for fine mapping of the TWAS signal associated with MASLD. In addition, transcriptome-wide significant gene-related top eQTLs emerged for many recurrent phenotypes associated with MASLD, such as triglyceride cholesterol and LDL cholesterol levels. Metabolic syndrome is prominently characterized by elevated triglycerides and low HDL-C levels, correlating with an elevated risk of MASLD.^30^ This aligns with the results of phenotype-wide association studies showing that certain eQTLs are strongly associated with MASLD.

Although the results obtained are promising, it is important to acknowledge certain limitations that merit attention. First, the correlations identified through TWAS may be subject to confounding factors, as the estimated gene levels are derived from a weighted linear combination of SNPs. These SNPs might be associated with non-modulatory factors that influence both correlation and risk, potentially leading to an overestimation of certain associations. Second, the TWAS approach in this study focused solely on the localized impacts of gene expression, neglecting the potential influence of distal effects. Future research should strive to construct larger gene expression reference frameworks to comprehensively examine local and distal effects.

## Conclusion

In summary, despite the limitations of our study, we present evidence of transcriptome-wide genetic variation in MASLD. Here, we have successfully identified several potentially significant genes related to MASLD, including MAU2, EPHA2, GATAD2A, and TM6SF2. In conclusion, TWAS represents a robust statistical tool for pinpointing effector genes associated with MASLD and advancing our comprehension of the disease’s molecular mechanisms.

## Supplementary Materials

Supplementary Material

## Figures and Tables

**Table 1.** Significant TWAS Genes for MASLD

Cytogenetic Band	TWAS Identified Genes	Panel	eQTL ID	BEST.GWAS.ID	TWAS Z Score	TWAS *P* Value	PP4	Implicated in Previous MASLD GWASs
19p13.11	*MAU2*	Whole_Blood	rs1859287	rs12610185	−8.25	1.57e−16	0.98	Yes
1p36.13	*EPHA2*	Liver	rs7538216	rs6677710	−5.06	4.25e−07	0.99	No
19p13.11	*GATAD2A*	Whole_Blood	rs4808203	rs12610185	4.73	2.29e−06	0	Yes
19p13.11	*TM6SF2*	Liver	rs4808200	rs12610185	−4.68	2.84e−06	0.39	Yes

eQTL, expression quantitative trait locus; GWAS, genome-wide association study; PP, posterior probability; TWAS, transcriptome-wide association study.

**Table 2.** Causal Posterior Probabilities for Genes in 90% Credible Sets for MASLD TWAS Signals with Z-Score >|6|

Block	Gene	Tissue	TWAS Z	Posterior Probability for Causality
22:43714200-22:44995308	*PNPLA3*	Skin sun exposed lower leg	−11.3	0.87
22:43714200-22:44995308	*PNPLA3*	Lung	6.81	0.87
19:18409862-19:19877471	*GATAD2A*	Oesophagus mucosa	8.83	0.65
8:126410917-8:128659111	*LINC00964*	Heart atrial appendage	−6.57	0.54
8:126410917-8:128659111	*TATDN1*	Muscle skeletal	6.55	0.45
22:43714200-22:44995308	*TTLL12*	Small intestine terminal ileum	11.4	0.45
19:18409862-19:19877471	*ZNF93*	Adrenal gland	−8.8	0.32
19:18409862-19:19877471	*ZNF90*	Skin sun exposed lower leg	−8.48	0.03
19:18409862-19:19877471	*LINC00663*	Small intestine terminal ileum	6.29	0.01
19:18409862-19:19877471	*ATP13A1*	Blood	6.03	0.00
19:18409862-19:19877471	*NCAN*	Brain cerebellum	7.03	0.00
19:18409862-19:19877471	*GATAD2A*	Whole blood	6.2	0.00
19:18409862-19:19877471	*GATAD2A*	Skin not sun exposed suprapubic	6.11	0.00
19:18409862-19:19877471	*LINC00663*	Minor salivary gland	7	0.00
19:18409862-19:19877471	*TM6SF2*	Heart left ventricle	6.12	0.00
19:18409862-19:19877471	*ATP13A1*	Nerve tibial	6.97	0.00
19:18409862-19:19877471	*MAU2*	Whole blood	−6.91	0.00
19:18409862-19:19877471	*ZNF486*	Lung	−6.05	0.00
19:18409862-19:19877471	*ATP13A1*	Adipose subcutaneous	6.88	0.00
19:18409862-19:19877471	*ATP13A1*	Artery coronary	6.93	0.00
19:18409862-19:19877471	*ATP13A1*	Spleen	6.98	0.00
19:18409862-19:19877471	*ATP13A1*	Artery aorta	6.69	0.00
19:18409862-19:19877471	*ATP13A1*	Skin sun exposed lower leg	6.71	0.00
19:18409862-19:19877471	*ATP13A1*	Esophagus muscularis	6.29	0.00
19:18409862-19:19877471	*ATP13A1*	Lung	6.66	0.00
19:18409862-19:19877471	*ATP13A1*	Artery tibial	6.27	0.00

**Table 3.** The Significant Probes Identified in the SMR Analysis

eQTL Data	CHR	Probe ID	Gene	Top SNP	*P*_eQTL_	*P*_GWAS_	Beta	SE	*P*_SMR_	*P*_HEIDI_	N_SNP_
*Westra et al*.	19	ILMN_1,780,987	*RFXANK*	rs11668319	2.43e−19	3.63e−09	0.84	0.17	8.29e−07	2.41e−05	11
	19	ILMN_1,685,631	*KIAA0892*	rs2301668	0.00e+00	2.30e−08	−0.18	0.03	2.88e−08	2.92e−11	20
	19	ILMN_1,673,682	*GATAD2A*	rs6909	5.12e−210	2.42e−06	0.22	0.05	3.07e−06	1.68e−08	20
	19	ILMN_2,134,224	*ATP13A1*	rs2304130	0.00e+00	2.81e−12	0.27	0.04	6.62e−12	8.00e−04	17
CAGE	19	ILMN_1,685,631	*MAU2*	rs11085261	1.19e−229	2.51e−08	−0.16	0.03	4.19e−08	4.03e−11	20
	19	ILMN_1,673,682	*GATAD2A*	rs4808203	2.31e−109	2.31e−06	0.19	0.04	3.70e−06	4.65e−09	20
	19	ILMN_2,134,224	*ATP13A1*	rs58434384	1.60e−119	2.24e−12	0.27	0.04	1.89e−11	2.72e−-04	20
GTE_X_ (whole_blood)	19	ENSG00000129933	*MAU2*	rs57962361	2.17e−15	1.51e−12	−1.7	0.32	1.28e-07	2.94e−02	20

CHR, chromosome;eQTL, expression quantitative trait locus; GWAS, genome-wide association study; HEIDI, heterogeneity in dependent instruments; SE, standard error; SMR, summary data-based Mendelian randomization; SNP, single nucleotide polymorphism.

**Supplementary Table 1.** Genomic risk loci detected from NAFLD GWAS results

https://docs.google.com/spreadsheets/d/1oMwzQtdBc8bbug1JgCO8z2OFzPcRgm44V-vIUTGsYDU/edit?usp=sharing

**Supplementary Table 2.** Lead SNPs identified from independent significant SNPs of NAFLD GWAS

https://docs.google.com/spreadsheets/d/1oMwzQtdBc8bbug1JgCO8z2OFzPcRgm44V-vIUTGsYDU/edit?usp=sharing

**Supplementary Table 3.** Independent significant SNPs identified from NAFLD GWAS

https://docs.google.com/spreadsheets/d/1oMwzQtdBc8bbug1JgCO8z2OFzPcRgm44V-vIUTGsYDU/edit?usp=sharing

**Supplementary Table 4.** Candidate SNPs identified from NAFLD GWAS

https://docs.google.com/spreadsheets/d/1oMwzQtdBc8bbug1JgCO8z2OFzPcRgm44V-vIUTGsYDU/edit?usp=sharing

**Supplementary Table 5.** Functional consequences of SNPs on genes

https://docs.google.com/spreadsheets/d/1oMwzQtdBc8bbug1JgCO8z2OFzPcRgm44V-vIUTGsYDU/edit?usp=sharing

**Supplementary Table 6.** Prioritized genes from NAFLD GWAS by functional mapping

https://docs.google.com/spreadsheets/d/1oMwzQtdBc8bbug1JgCO8z2OFzPcRgm44V-vIUTGsYDU/edit?usp=sharing

**Supplementary Table 7.** FUMA annotation pathway categories

https://docs.google.com/spreadsheets/d/1oMwzQtdBc8bbug1JgCO8z2OFzPcRgm44V-vIUTGsYDU/edit?usp=sharing

**Table 4.** Phenotypes Associated with Genes Derived from TWAS

TWAS Identified Genes	PMID	Year	Domain	Trait	*P*-value	N
*MAU2*	27863252	2016	Immunological	Plateletcrit (three-way meta)	1.77E−24	164 339
	27863252	2016	Immunological	Plateletcrit (two-way meta)	2.50E−24	127 033
	30124842	2018	Skeletal	Height	2.23E−22	693 529
	27863252	2016	Immunological	Red cell distribution width (two-way meta)	1.43E−17	131 520
	24097068	2013	Metabolic	Triglycerides cholesterol	4.74E−17	188 577
	24097068	2013	Metabolic	Total cholesterol	1.05E−16	188 577
	31427789	2019	Skeletal	Standing height	1.55E−15	385 748
	27863252	2016	Immunological	Red cell distribution width (three-way meta)	6.08E−15	171 529
	24097068	2013	Metabolic	Low-density lipoprotein cholesterol	1.59E−14	188 577
	27863252	2016	Immunological	Platelet count (three-way meta)	4.49E−14	166 066
*EPHA2*	29403010	2018	Metabolic	Gamma-glutamyl transferase	3.40E−11	118 309
	31427789	2019	Skeletal	Standing height	1.41E− 09	385 748
	31427789	2019	Metabolic	Impedance measures − Leg fat-free mass (right)	8.54E−08	379 793
	31427789	2019	Metabolic	Impedance measures − Whole body water mass	9.79E−08	379 835
	31427789	2019	Metabolic	Impedance measures − Whole body fat-free mass	9.88E−08	379 804
	31427789	2019	Metabolic	Impedance measures − Leg predicted mass (right)	1.01E−07	379 793
	31427789	2019	Metabolic	Impedance measures − Trunk fat-free mass	1.53E−07	379 507
	31427789	2019	Metabolic	Impedance measures − Basal metabolic rate	1.54E−07	379 821
	31427789	2019	Metabolic	Impedance measures − Trunk predicted mass	1.63E−07	379 469
	31427789	2019	Metabolic	Impedance measures − Arm predicted mass (left)	1.09E−06	379 638
*GATAD2A*	27863252	2016	Immunological	Plateletcrit (two-way meta)	5.44E−24	127 033
	27863252	2016	Immunological	Plateletcrit (three-way meta)	4.69E−23	164 339
	30124842	2018	Skeletal	Height	7.83E−16	693 529
	24097068	2013	Metabolic	Triglycerides cholesterol	1.02E−15	188−577
	24097068	2013	Metabolic	Total cholesterol	1.91E−15	188 577
	27863252	2016	Immunological	Red cell distribution width (two-way meta)	8.50E−15	131 520
	31427789	2019	Skeletal	Standing height	2.30E−14	385 748
	27863252	2016	Immunological	Red cell distribution width (three-way meta)	3.78E−14	171 529
	24097068	2013	Metabolic	Low-density lipoprotein cholesterol	4.79E−14	188 577
	27863252	2016	Immunological	Platelet count (two-way meta)	5.73E−14	127 127
*TM6SF2*	30124842	2018	Skeletal	Height	1.68E−24	693 529
	31217584	2019	Metabolic	Triglyceride	1.23E−23	33 096
	27863252	2016	Immunological	Plateletcrit (two-way meta)	1.25E−20	127 033
	31427789	2019	Metabolic	Impedance measures − Impedance of whole body	3.21E−20	379 792
	27863252	2016	Immunological	Plateletcrit (three-way meta)	4.35E−20	164339
	31427789	2019	Metabolic	Impedance measures - Impedance of arm (left)	1.65E−19	379 803
	31427789	2019	Metabolic	Impedance measures - Impedance of arm (right)	6.04E−19	379 786
	24097068	2013	Metabolic	Triglycerides cholesterol	1.11E−15	188 577
	24097068	2013	Metabolic	Total cholesterol	8.57E−15	188 577
	30124842	2018	Skeletal	Height	1.68E−24	693 529

## Data Availability

The data that support the findings of this study are available on request from the corresponding author. FUSION (http://gusevlab.org/projects/fusion/#gtex-v8-multi-tissue-expression) **Ethics Committee Approval:** N/A.
