# Supplementary material for: Transcriptome-Wide Association Study of Metabolic Dysfunction-Associated Steatotic Liver Disease Identifies Relevant Gene Signatures
Source: Turk J Gastroenterol. 2024 Dec 23;36(5):280–92. doi: 10.5152/tjg.2024.24326 (PMC12070431; doi:10.5152/tjg.2024.24326)
Supplement: Supplementary Material [file supplementary_material.pdf]

Supplementary Table 1. Genomic risk loci detected from NAFLD GWAS results

https://docs.google.com/spreadsheets/d/1oMwzQtdBc8bbug1JgCO8z2OFzPcRgm44V-vIUTGsYDU/edit?usp=sharing

Supplementary Table 2. Lead SNPs identified from independent significant SNPs of NAFLD GWAS

https://docs.google.com/spreadsheets/d/1oMwzQtdBc8bbug1JgCO8z2OFzPcRgm44V-vIUTGsYDU/edit?usp=sharing

Supplementary Table 3. Independent significant SNPs identified from NAFLD GWAS

https://docs.google.com/spreadsheets/d/1oMwzQtdBc8bbug1JgCO8z2OFzPcRgm44V-vIUTGsYDU/edit?usp=sharing

Supplementary Table 4. Candidate SNPs identified from NAFLD GWAS

https://docs.google.com/spreadsheets/d/1oMwzQtdBc8bbug1JgCO8z2OFzPcRgm44V-vIUTGsYDU/edit?usp=sharing

Supplementary Table 5. Functional consequences of SNPs on genes

https://docs.google.com/spreadsheets/d/1oMwzQtdBc8bbug1JgCO8z2OFzPcRgm44V-vIUTGsYDU/edit?usp=sharing

Supplementary Table 6. Prioritized genes from NAFLD GWAS by functional mapping

https://docs.google.com/spreadsheets/d/1oMwzQtdBc8bbug1JgCO8z2OFzPcRgm44V-vIUTGsYDU/edit?usp=sharing

Supplementary Table 7. FUMA annotation pathway categories

https://docs.google.com/spreadsheets/d/1oMwzQtdBc8bbug1JgCO8z2OFzPcRgm44V-vIUTGsYDU/edit?usp=sharing
